# Aberrant CD4 expression in plasmablastic transformation of multiple myeloma

**DOI:** 10.1002/jha2.935

**Published:** 2024-05-21

**Authors:** Kohei Shiroshita, Sumiko Kohashi, Miki Sakamoto, Himari Kudo, Kuniaki Nakanishi, Takaaki Toyama

**Affiliations:** ^1^ Department of Hematology Federation of National Public Service Personnel Mutual Aid Associations Tachikawa Hospital Tokyo Japan; ^2^ Division of Hematology, Department of Medicine Keio University School of Medicine Tokyo Japan; ^3^ Department of Pathology Federation of National Public Service Personnel Mutual Aid Associations Tachikawa Hospital Tokyo Japan

**Keywords:** histological transformation, myeloma, plasmablastic myeloma

1

A 68‐year‐old female patient with a 5‐year history of λ‐type IgD plasmacytoma was admitted to our hospital for the evaluation of fatigue in September 2023. The diagnosis of plasmacytoma was confirmed through a biopsy of the bone mass lesion, revealing mature plasma cells secreting monoclonal λ‐type IgD (elevated at 13.1 mg/dL). After autologous hematopoietic stem cell transplantation following bortezomib, lenalidomide, and dexamethasone treatment, the patient achieved stringent complete remission (CR) and continued lenalidomide maintenance therapy. In May 2022, daratumumab, carfilzomib, and dexamethasone were administered for paraprotein relapse. Upon admission, the laboratory findings revealed anemia (hemoglobin: 7.3 g/dL), hypercalcemia (corrected Ca: 13.5 mg/dL), and acute kidney injury (creatinine: 3.0 mg/dL). IgD was detected at low levels (1.4 mg/dL), and another immunoglobulin was suppressed. Urine analysis revealed λ‐type Bence Jones protein (BJP) at 2271 mg/day. She was not infected with the human immunodeficiency virus. Bone marrow biopsy revealed sheets of atypical cells with plasmablastic morphology, located centrally, with prominent nuclei and a high nuclear‐to‐cytoplasmic ratio (Figure 1, left image). The cells were also positive for CD138 (Figure 1, middle image), CD4 (Figure 1, right image), and cyclin D1, and negative for c‐MYC, EBER, CD3, CD8, CD34, TdT, CD20, and CD79a. The Ki‐67 index was 80%. Fluorescence in situ hybridization identified 1q gain, del 17p, and *IgH::CCND1*, and the chromosomes had a complex karyotype. Based on these findings, the patient was diagnosed with triple‐class exposed BJP‐λ‐type plasmablastic myeloma (PBM) with aberrant CD4 expression. Although she achieved CR after two courses of cyclophosphamide, adriamycin, vincristine, and prednisolone (CHOP), the disease relapsed after the third CHOP cycle. The patient refused additional therapy and was shifted to the best supportive care.

**FIGURE 1:**
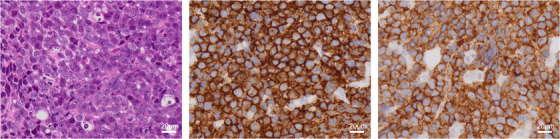
The results of bone marrow biopsy at diagnosis of plasmablastic myeloma: hematoxylin‐eosin staining (left), CD138 staining (middle), and CD4 staining (right).

PBM is an aggressive multiple myeloma subtype, with a poor prognosis. In addition to morphological transformation, aberrant T‐cell marker expression is useful for evaluating disease prognosis [1]. Although new‐class drugs and cytotoxic chemotherapy enable disease control in PBM, early treatment resistance in PBM with aberrant CD4 expression remains a clinical challenge. Novel consolidative therapies, including chimeric antigen receptor T cells and bispecific antibodies, are worth considering for such patients.

## AUTHOR CONTRIBUTIONS

Kohei Shiroshita designed the study and collected the clinical data. Kohei Shiroshita, Sumiko Kohashi, Miki Sakamoto, Himari Kudo, and Takaaki Toyama treated enrolled patients. Kuniaki Nakanishi performed pathological analyses. Kohei Shiroshita wrote the manuscript. Kohei Shiroshita. and Takaaki Toyama supervised manuscript preparation. All authors contributed to the drafting of the manuscript and approved its submission.

## CONFLICT OF INTEREST STATEMENT

No conflicts of interest were declared.

## ETHIC STATEMENT

The authors have confirmed ethical approval statement is not needed for this submission.

## PATIENT CONSENT STATEMENT

Informed consent for the publication of this article was obtained from all enrolled patients. This study did not require institutional ethics committee approval.

## CLINICAL TRIAL REGISTRATION

The authors have confirmed clinical trial registration is not needed for this submission.

## Data Availability

Data sharing does not apply to this article as no new data were created.
